# Gypenoside XLIX ameliorates diabetic retinopathy by downregulating prostaglandin-endoperoxide synthase 2 in retinal pigment epithelium cells to inhibit ferroptosis and preserve tight junction integrity

**DOI:** 10.3389/fphar.2026.1777313

**Published:** 2026-03-23

**Authors:** Jiayi Gu, Manhui Zhu, Lele Li, Xin Cao, Xiaoli Yu, Xiaobo Huang, Lili Huang, Qi Cai, Yan Zhu, Wendie Li, Yong Wang

**Affiliations:** 1 Ophthalmology Department, Nantong First People’s Hospital, Southeast University, Nantong, Jiangsu, China; 2 Department of Ophthalmology, Lixiang Eye Hospital of Soochow University, Suzhou, Jiangsu, China; 3 Department of Ophthalmology, Ningbo Eye Hospital, Ningbo, Zhejiang, China

**Keywords:** diabetic retinopathy, ferroptosis, gypenoside XLIX, networkpharmacology, prostaglandin-endoperoxide synthase 2, retinal pigment epithelium

## Abstract

**Introduction:**

Diabetic retinopathy (DR) represents a prevalent and severe eye complication in diabetic patients. With DR progresses, destruction of tight junctions (TJs) in RPE cells leads to irreversible visual impairment. Gypenoside XLIX (Gyp XLIX) is a dammarane-type glycoside, which can suppress inflammation and oxidative stress. This study sought to investigate and verify the mechanism underlying the regulatory effects of Gyp XLIX in the early protection of junctional integrity of DR.

**Methods:**

We combined bioinformatics and network pharmacology to pinpoint the core therapeutic targets of Gyp XLIX for DR. Mice with diabetes mellitus (DM) and high glucose (HG)-stimulated ARPE-19 cells were treated with Gyp XLIX. Its impact on TJ integrity in RPE cells and ferroptosis was evaluated via Western blotting, immunofluorescence staining, and assays for iron content, lipid peroxidation, and glutathione (GSH) levels. Prostaglandin-endoperoxide synthase 2 (PTGS2) was overexpressed to elucidate the mechanism of action of Gyp XLIX in the early protection of junctional integrity of DR.

**Results:**

Among the shared targets between Gyp XLIX and DR, ALB, VEGFA, JUN, ESR1, PTGS2, STAT3, MMP9, HSP90AA1, BCL2L1 and AR were identified. Western blotting and immunofluorescence staining revealed that Gyp XLIX preserved TJ integrity in RPE cells. In addition, iron, lipid peroxidation and GSH assays revealed that Gyp XLIX inhibited ferroptosis in both mice with DM and HG-stimulated ARPE-19 cells. Overexpression of PTGS2 partially reversed the protective impacts induced by Gyp XLIX.

**Discussion:**

This study demonstrated that Gyp XLIX suppressed ferroptosis and preserved TJ integrity in RPE cells, with these effects being closely associated with the downregulation of PTGS2, thereby exerting early protective effects on junctional integrity of DR.

## 1 Introduction

Diabetic retinopathy (DR) represents a prevalent and serious eye complication in diabetic patients, and it is the primary cause of vision loss in middle-aged individuals in industrialized countries worldwide (Levine et al., 2022). In China, more than 100 million individuals have diabetes, and the incidence of DR among adult patients with diabetes ranges from 24.7% to 37.5%, with the number of patients exceeding 30 million. Previous studies have revealed that various metabolic pathways, including the polyol pathway, protein kinase C activation, oxidative stress and endoplasmic reticulum stress, contribute to the development and progression of DR (Roy et al., 2017; Santos et al., 2011). Currently available DR treatments, include laser photocoagulation, intravitreal injection of anti-vascular endothelial growth factor (anti-VEGF) antibodies or glucocorticoids, and surgery face several challenges, such as nonresponsiveness in some patients, poor long-term visual improvement, the heavy economic burden associated with repeated intravitreal injections, and adverse side effects (Campochiaro and Akhlaq, 2021). Good glycemic control can inhibit the development and progression of DR, but maintaining it remains challenging for many patients (Stitt et al., 2016). Therefore, more effective and multitarget therapeutic strategies are required to control the onset and progression of DR.

DR is a neurovascular disease characterized by neuronal and vascular abnormalities (Rossino, Dal Monte, and Casini, 2019). The underlying mechanisms contributing to vascular dysfunction include modifications in the blood-retinal barrier (BRB), which lead to subretinal and intraretinal fluid accumulation, causing diabetic macular edema (DME) (Daruich et al., 2018; O'Leary and Campbell, 2023). In recent years, the impact of hyperglycemia on retinal pigment epithelium (RPE) cells, major components of the outer blood-retinal barrier (oBRB), has received increased attention. Studies have shown that tight junctions (TJs) in the RPE play an essential role in forming the oBRB (Konari et al., 1995). The TJ proteins and TJ-related proteins that have been confirmed to contribute to oBRB formation include zonula occludens-1 (ZO-1), occludin and others (Furuse et al., 1993; Stevenson et al., 1986). The impairment of TJs in RPE cells caused by hyperglycemia and oxidative stress is a critical cause of early DR. Thus, targeting the restoration of TJ function in RPE cells has emerged as a promising strategy for treating DR.

In recent years, researchers have discovered that ferroptosis, an emerging form of regulated cell death (RCD), may be crucial in the pathogenesis of DR (You et al., 2022). Ferroptosis is caused by iron-dependent buildup of lipid peroxides (Tang D. et al., 2021; Tang W. et al., 2021), which is important for various diseases, including cancer and neurodegenerative diseases (Mou et al., 2019). The classical glutathione (GSH)–glutathione peroxidase 4 (GPX4) axis is responsible for regulating ferroptosis (Proneth and Conrad, 2019). In addition, ferroptosis suppressor protein-1 (FSP-1) is an effective inhibitor of ferroptosis (Doll et al., 2019). Solute carrier family 7 member 11 (SLC7A11), a cell membrane protein, is one of the subunits of system Xc-, which is a cystine–glutamate antiporter that is one of the primary regulatory systems of lipid peroxidation during ferroptosis (Chaudhary et al., 2018; Shao et al., 2022). In addition, Hjv^−/−^ mice, a model of genetic iron overload, have been reported to exhibit breakdown of the BRB, which is attributed to decreased expression of TJ proteins (Tawfik et al., 2014). Thus, it is hypothesised that ferroptosis could represent a viable therapeutic target for DR.

Traditional Chinese Medicine (TCM) has shown efficacy in preventing DR through replenishing Qi and nourishing Yin while concomitantly clearing heat and promoting fluid production, thereby activating blood circulation and eliminating blood stasis (Ai et al., 2020; Xu et al., 2018). Ethnopharmacologically, *Gynostemma pentaphyllum* (*G. pentaphyllum*), a Chinese medicinal herb, has been revered for centuries in East and Southeast Asia, particularly in China, Japan, and Vietnam, as a folk remedy for a wide spectrum of ailments (Chen et al., 1996). In TCM, it is characterized as sweet, slightly bitter, and neutral, with the ability to enhance “yin” and support “yang”, making it a versatile adaptogenic herb used for hyperlipidemia, palpitation, shortness of breath, chest congestion, dizziness, headache, forgetfulness, tinnitus, general weakness, and abdominal swelling (Nguyen et al., 2021). Studies have demonstrated that *G. pentaphyllum* exerts multiple therapeutic properties, including anti-inflammatory (Wang et al., 2017), antioxidant (Zhang et al., 2011), anti-cancer (Liu et al., 2014) and neural protection (Wang et al., 2022) effects. The pharmacological activities of *G. pentaphyllum* are attributed to its dammarane-type triterpene gypenosides to a significant extent (Li et al., 2019). Gypenoside XLIX (Gyp XLIX), which is a dammarane-type glycoside, is the main saponin of *G. pentaphyllum* (Guo et al., 1997). It can suppress renal inflammation and fibrosis by selectively activating peroxisome proliferator-activated receptor-alpha (PPAR-α) (Liu et al., 2021; Yang Q. et al., 2021), and alleviate oxidative stress and inflammation through suppression of nuclear factor-kappa B (NF-κB) activation (Chen et al., 2022; Huang et al., 2006). Therefore, we hypothesized that Gyp XLIX might have potential for the therapeutic management of DR.

In this work, we adopted a multifaceted approach including bioinformatics, systems biology and comprehensive network pharmacology to clarify the potential mechanism underlying the therapeutic effects of Gyp XLIX on DR (Hopkins, 2008). A total of 10 key hub genes were recognized, including the ferroptosis-related target prostaglandin-endoperoxide synthase 2 (PTGS2). Both *in vivo* animal models and *in vitro* cell-based studies were executed to assess whether Gyp XLIX regulated ferroptosis to exert early protective effects on junctional integrity of DR, and to explore the involvement of PTGS2 in this process. This study provides a theoretical and experimental reference for the subsequent clinical implementation of Gyp XLIX in the treatment of DR.

## 2 Materials and methods

### 2.1 Bioinformatics analysis

The 2D molecular structure of Gyp XLIX was retrieved from the PubChem database. Gyp XLIX targets were predicted using PharmMapper and SwissTargetPrediction. DR-associated targets were derived from the GSE53257 dataset and from GeneCards, OMIM, and DisGeNET. The GSE53257 dataset contained 16 neural retina specimens obtained from postmortem eyes, consisting of 5 samples from normal controls, 5 samples from patients with diabetes mellitus (DM), and 6 samples from patients diagnosed with DR. GPL18056, a custom-designed human 8 × 15K microarray platform (AMADID: 045815) developed by Genotypic Technology Private Limited, was utilised to analyse GSE53257. Differentially expressed genes (DEGs) between the retina samples of healthy individuals and patients with DR were identified using the Microarray Data Linear Module Bioconductor (Robinson et al., 2010). A volcano map and heatmap visualizing the DEGs were created using the R programming language. Overlapping targets between Gyp XLIX and DR were submitted to STRING and the protein–protein interaction (PPI) network was constructed and analysed in Cytoscape with cytoHubba. Kyoto Encyclopedia of Genes and Genomes (KEGG) and Gene Ontology (GO) enrichment analyses were carried out to elucidate the biological roles of the composite targets. High-resolution crystal structures of the Gyp XLIX active components, along with their bioactive ligands were obtained from the Protein Data Bank (PDB). Molecular docking of Gyp XLIX to target proteins was conducted after ligand removal and dehydration in PyMOL. Molecular docking simulations were performed based on the ligand coordinates in the target protein complex and the size of the grid box according to the protein’s active pocket. AutoDock Vina was used to perform the docking. The complete database versions, parameters, and deduplication rules were shown in Supplementary Table S1.

### 2.2 Chemicals and reagents

Gyp XLIX (CAS# 94987-08-3, ≥98% purity confirmed by high-performance liquid chromatography) was acquired from Aladdin (Shanghai, China; Cat# G414354). Streptozotocin (STZ; CAS# 18883-66-4) was sourced from Macklin (Shanghai, China; Cat# S817944). Cell culture reagents including Dulbecco’s Modified Eagle Medium (DMEM; high glucose with L-glutamine; Gibco, Cat# 11965118), fetal bovine serum (FBS; Gibco, Cat# 12483020), and penicillin-streptomycin solution (10,000 U/mL penicillin, 10 mg/mL streptomycin; Gibco, Cat# 15140122) were purchased from Thermo Fisher Scientific (Waltham, MA, United States). MTT (3-(4,5-dimethylthiazol-2-yl)-2,5-diphenyltetrazolium bromide) reagent (Cat# 88417) was sourced from Sigma-Aldrich (Shanghai, China). A TRIzol reagent kit (Cat# 16096040) was sourced from Invitrogen (Carlsbad, CA, United States). RIPA lysis buffer (Cat# RFZ-RP-5024) was sourced from Beyotime (Shanghai, China). Protease and phosphatase inhibitor cocktails (Cat# IKM1020) were sourced from Solarbio (Beijing, China). A BCA protein assay kit (Cat# JX00600,379), a lipid peroxidation assay kit (Cat# S0131S) and a GSH assay kit (Cat# S0052) were acquired from Beyotime (Shanghai, China). Polyvinylidene fluoride (PVDF) membranes (Cat# IPVH00010) were sourced from Millipore (Burlington, MA, United States). An iron assay kit (Cat# ab83366) was purchased from Abcam (Cambridge, United Kingdom). The antibodies used in this study included: TUBULIN (1:2000; Cat# ab6046), β-actin (1:2000; Cat# ab8227), anti-ZO-1 (1:1,000; Cat# ab276131), anti-occludin (1:1,000; Cat# ab216327), anti-GPX4 (1:1,000; Cat# ab125066) and PTGS2 (1:2000; Cat# ab179800) from Abcam (Cambridge, United Kingdom); FSP-1 (1:1,000; Cat# 24972) from Affinity Biosciences (Jiangsu, China); SLC7A11 (1:1,000; Cat# 26864-1-AP) from Proteintech (Rosemont, IL, United States). Anti-ZO-1 (1:100; Cat# ab221547) and 4′,6-diamidino-2-phenylindole (DAPI; 1:5,000; Cat# ab228549) from Abcam (Cambridge, United Kingdom).

### 2.3 Animal grouping and treatment

Male C57BL/6 mice (6–8 weeks old, weighing approximately 20 g on average) were purchased from the Laboratory Animal Center of Nantong University. Only male mice were used, as STZ-induced hyperglycemia is more stable and reproducible in males, consistent with established literature in this field. All animal experimental procedures were approved by the Animal Research Ethics Committee of Nantong University and conducted in accordance with the ARVO Statement for the Use of Animals in Ophthalmic and Vision Research. Mice were housed under controlled ambient conditions (22 °C ± 1 °C, 50% ± 10% humidity, 12 h light/dark cycle) with free access to food and water; all procedures were performed between 9:00 and 11:00 AM to minimize circadian variation. We minimized animal suffering and applied humane endpoints to safeguard mouse welfare throughout the study; predefined humane endpoints included body weight loss exceeding 20% from baseline, persistent hypothermia, or inability to reach food or water, none of which were reached during the experiment. The mice were randomly assigned to experimental groups using a random number table generated by an investigator not involved in animal handling, and allocation concealment was ensured by sequentially numbered, opaque, sealed envelopes. All outcome assessments were performed by investigators blinded to group allocation; image files were coded and analyzed in random order to eliminate subjective bias. Mice in the DR group were maintained on a high-fat diet (60 kcal% fat, 20 kcal% carbohydrate, 20 kcal% protein, with a total energy of 5.24 kcal/gm; Ca#D12492, Research Diets, Inc., United States) and intraperitoneally injected with 50 mg/kg/d STZ for 5 days. Mice in the control group were maintained on a normal diet and administered citric acid buffer at the same does and time as STZ. After 7 days of acclimatization, mice with consistently elevated blood glucose levels (i.e., >16.7 mmol/L) were selected for investigation. The baseline parameters of the diabetes model, including blood glucose levels, body weight changes, and the duration of hyperglycemia, were shown in Supplementary Figure S1. After 8 weeks of the successful establishment of the model, the mice were treated with Gyp XLIX (50 mg/kg/day) for 7 days via intraperitoneal injection. At this dosage, the compound demonstrated a favorable safety profile with no mortality or treatment-related toxicity observed throughout the study period. After 7 days, the mice were sacrificed, and their eyeballs were harvested for subsequent experimental procedures. For *in vivo* PTGS2 overexpression, recombinant adeno-associated virus serotype 8 (AAV8) carrying the full-length mouse *Ptgs2* coding sequence (NM_011198.5) under the control of the CMV promoter was packaged and purified by GeneCopoeia (Rockville, MD, United States). The viral titer, determined by qPCR, was 2.5 × 10^13^ vg/mL, with endotoxin level <1 EU/mL. The mice were anesthetized and their pupils were dilated. Under a stereomicroscope, a 33-gauge Hamilton syringe (Hamilton, Cat# 61-0230) was inserted through the sclera at the pars plana into the vitreous cavity, and 1 μL of AAV8-CMV-PTGS2 (2.5 × 10^10^ vg) was slowly injected into the right eye. The contralateral eye received the same volume and titer of AAV8-CMV-GFP as control. The needle was held in place for 30 s after injection and then withdrawn slowly to minimize reflux. After injection, erythromycin ophthalmic ointment was applied to prevent infection and corneal desiccation. Mice were euthanized 14 days post-injection, and RPE/choroid complexes were collected for downstream analyses.

### 2.4 Cell culture and *in vitro* cell model of DR

The human RPE cell line ARPE-19 (CRL-2302) from American Type Culture Collection (ATCC, United States), which is Spontaneously immortalized epithelial cell lines retaining the morphological and functional characteristics of natural RPE cells. The cells were cultured in DMEM–10% FBS–penicillin/streptomycin (100 U/mL) at 37 °C. All experiments were conducted within 3–9 passages of cell type. The cells were assigned to the following experimental groups: normal glucose (NG, 5.5 mmol/L D-glucose medium), NG + mannitol, high glucose (HG, 30 mmol/L D-glucose medium), HG + oe-NC, HG + oe-PTGS2, HG + Gyp XLIX (50 μmol/L for 18 h), and HG + Gyp XLIX + oe-PTGS2. The oe-NC and oe-PTGS2 plasmids were supplied by GenePharma Co., Ltd. (Shanghai, China). Cells in the HG + Gyp XLIX + oe-PTGS2 groups were transfected with oe-PTGS2 (2 μg/mL) prior to Gyp XLIX treatment.

### 2.5 MTT assay

Cell viability was assessed using the MTT colorimetric assay. ARPE-19 cells were plated in 96-well plates and exposed to Gyp XLIX (0, 10, 25, 50, 100 and 200 μmol/L) for 18 h, and then MTT reagent (5 mg/mL) was supplemented. The optical density (OD) value at 492 nm was determined to evaluate cell viability.

### 2.6 Quantitative reverse transcription polymerase chain reaction (qRT-PCR)

Total mRNA was isolated using a TRIzol reagent. The quality and quantity of RNA was assessed using an Eppendorf BioSpectrometer (Eppendorf, Germany). Subsequently, 1 μg of the extracted RNA was reverse transcribed under the following conditions: 37 °C for 15 min, 85 °C for 5 min, and 4 °C. A 20 μL reaction mixture was prepared to amplify the synthesized cDNA (96 °C for 5 min and 60 °C for 30 s × 40 times). The 2^−ΔΔCT^ method was applied to quantify the expression levels of target gens. The primer sequences were shown in Table 1.

**TABLE 1:** The sequences of PCR primers used in the study was shown.

Name	Primer sequence (forward, 5′-3′)	Primer sequence (reverse, 5′-3′)
Mouse ZO-1	ACC​AGT​AAG​TCG​TCC​TGA​TCC	TCG​GCC​AAA​TCT​TCT​CAC​TCC
Mouse occludin	GAC​TTC​AGG​CAG​CCT​CGT​TAC	GCC​AGT​TGT​GTA​GTC​TGT​CTC​A
Human ZO-1	GAA​ATA​CCT​GAC​GGT​GCT​GC	GAG​GAT​GGC​GTT​ACC​CAC​AG
Human occludin	CTCCCTGGCACCGTTGG	TAC​AAT​GGC​AAT​GGC​CTC​CT
Mouse ALB	CCC​ACT​AGC​CTC​TGG​CAA​AA	TCA​CAC​CAT​CAA​GCT​TCG​GG
Mouse VEGFA	TTC​CCT​CTG​GGT​AGG​GAA​CC	CAG​AGG​AGG​CAC​CTT​TCA​GG
Mouse ESR1	AAG​ACG​CTC​TTG​AAC​CAG​CA	TCT​TTC​CGT​ATG​CCG​CCT​TT
Mouse PTGS2	CAT​CCC​CTT​CCT​GCG​AAG​TT	CCT​CTC​CAC​CAA​TGA​CCT​GAT
Mouse STAT3	TGC​CAA​TTG​TGA​TGC​CTC​CT	GTC​TTC​AGG​TAC​GGG​GCA​G
Mouse MMP9	AAA​CCT​CCA​ACC​TCA​CGG​AC	GTA​AGT​GGG​GAT​CAC​GAC​GC
Mouse HSP90AA1	CGA​CGA​TGA​GCA​GTA​TGC​CT	CGA​CCC​ATT​GGT​TCA​CCT​GT
Mouse BCL2L1	GCC​GGA​GAT​AGA​TTT​GAA​TAA​CCT	CTG​GTA​GCA​ATG​GTG​GCT​GA
Mouse β-actin	CCA​GCC​TTC​CTT​CTT​GGG​TAT	GGG​TGT​AAA​ACG​CAG​CTC​AG
Human β-actin	GCC​GGG​ACC​TGA​CTG​ACT​AC	CGG​ATG​TCC​ACG​TCA​CAC​TT

### 2.7 Western blotting

Protein extracts were obtained from mouse RPE/choroid complexes or ARPE-19 cells. The RPE/choroid complexes were homogenized in ice-cold RIPA lysis buffer containing protease and phosphatase inhibitor cocktails at a ratio of 100 mg tissue per 1 mL buffer (1:10 w/v). Homogenates were incubated on ice for 30 min and centrifuged at 12,000×g for 20 min at 4 °C. The supernatant was collected, and a BCA protein assay kit was performed to quantify the extracted protein. Equal amounts of protein (30 μg per lane) were separated via sodium dodecyl sulfate–polyacrylamide gel electrophoresis (SDS-PAGE, 10% gel) and subsequently transferred to PVDF membranes. After non-specific binding sites were blocked, the membrane was incubated with primary antibodies (prepared in 5% BSA) for 24 h at 4 °C. Autoradiograms were quantified with Quantity One software (Bio-Rad, United States). Band intensities of target proteins were first normalized to those of the corresponding loading control (β-actin or TUBULIN), and then normalized to the mean value of the control group, which was set to 1.0. All experiments were repeated at least three times with independent biological replicates.

### 2.8 Immunofluorescence staining

After preparing the paraffin sections of mouse eyeballs, the samples were incubated with 3% H_2_O_2_ for 15 min. For antigen retrieval, the samples were microwave-heated in sodium citrate buffer and allowed to cool. Subsequently, the samples were incubated with anti-ZO-1, stained with DAPI, and sealed. The sections were imaged with an Olympus confocal laser scanning microscope (FV 1000, Olympus), and the fluorescence intensity per unit area of ZO-1 was calculated using the manufacturer’s analytical software.

### 2.9 Iron assay

Mouse RPE/choroid complexes were washed with pre-chilled PBS and homogenised. Intracellular ferrous iron (Fe^2+^) levels in the tissues were determined using an iron assay kit. The absorbance at 520 nm was recorded with a multifunctional enzyme labeller (BioTek Synergy HTX). The assay was performed in triplicate. ARPE-19 cells were incubated with Ferro Orange (1 μmol/L) at 37 °C. The medium was replaced with serum-free DMEM, and the cells were then visualized under an Olympus microscope, and the images were analysed with ImageJ.

### 2.10 Lipid peroxidation and GSH assays

To assess lipid peroxidation levels, malondialdehyde (MDA) and GSH levels were evaluated in ARPE-19 cells and mouse RPE/choroid complexes using a lipid peroxidation assay kit and a GSH assay kit, respectively.

### 2.11 Statistical analysis

All data were processed with the GraphPad Prism (version 9.0) software. Data are presented as mean ± standard deviation (SD), with each experimental group including a minimum of three independent biological replicates; technical replicates were averaged prior to statistical analysis. For comparisons between two groups, a two-tailed unpaired Student’s t-test was used. For multiple group comparisons, one-way or two-way ANOVA was applied, followed by Tukey’s *post hoc* test for pairwise comparisons, with homogeneity of variances assessed using Brown–Forsythe and Bartlett’s tests. When variance equality was not met, appropriate variance-corrected ANOVA methods were applied. To control for multiple testing, the Benjamini–Hochberg false discovery rate (FDR) procedure was applied with a 5% threshold. Effect sizes were calculated as Cohen’s d (for t-tests) or partial η^2^ (for ANOVA), and their 95% confidence intervals were estimated using bias-corrected bootstrap. A p-value of <0.05 was considered to indicate statistical significance, and non-significant trends are not interpreted or discussed.

## 3 Results

### 3.1 Identification and validation of core targets of gyp XLIX against DR

**FIGURE 1:**
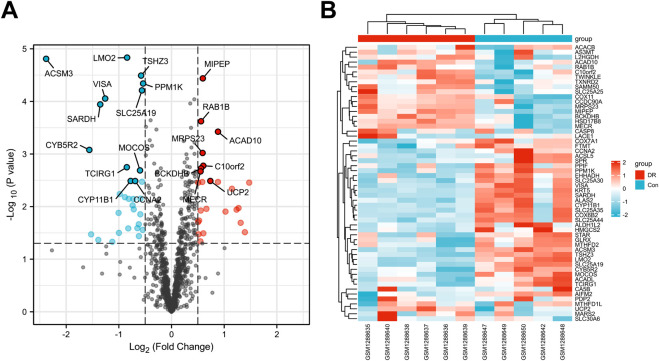
Prediction of targets of Gyp XLIX and diabetic retinopathy (DR). **(A)** Volcano map demonstrating the top 20 differentially expressed genes between the normal and DR groups in the GSE53257 dataset, with red dots representing upregulated genes and blue dots representing downregulated genes. **(B)** Heat map of differentially expressed genes (DEGs) demonstrating upregulated (red) and downregulated (blue) genes in order of their false discovery rate (FDR) values from low to high.

**FIGURE 2:**
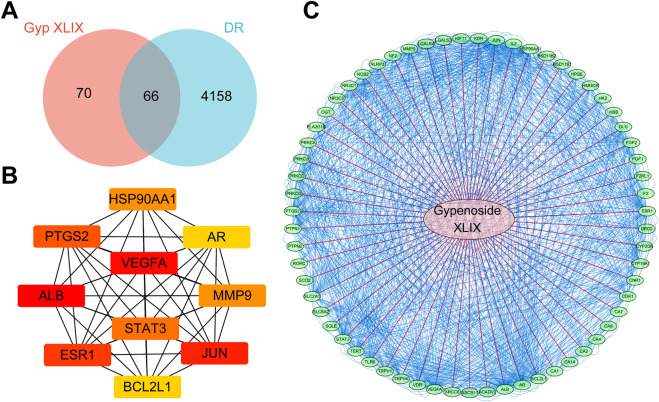
Identification and analysis of core targets of Gyp XLIX for the treatment of diabetic retinopathy (DR). **(A)** Intersection of the targets of Gyp XLIX and DR. The blue and red ellipses represent the predicted targets of DR and Gyp XLIX, respectively, and the middle part represents the common targets of Gyp XLIX and DR. **(B)** Protein–protein interaction (PPI) networks were visually analysed using the Cytoscape (version 3.7.2) software. **(C)** The top 10 genes with the highest degree values. Each node represents a protein, and each edge represents the relationship between the proteins.

**FIGURE 3:**
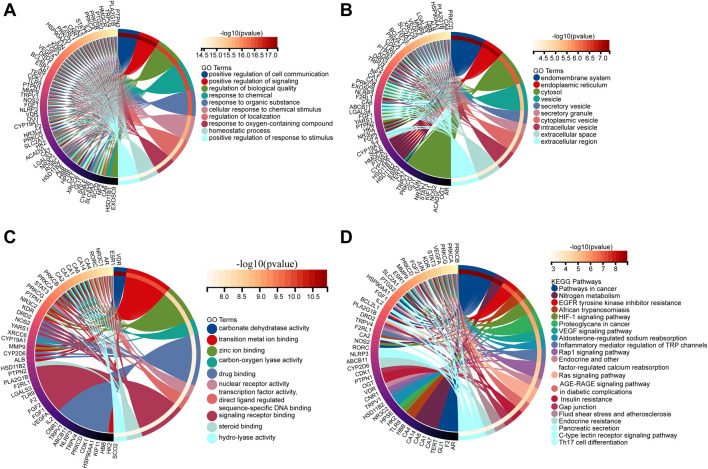
Functional enrichment analysis of hub Genes. Gene Ontology (GO) enrichment analysis of composite target genes: BPs **(A)**, CCs **(B)** and MFs **(C)**. **(D)** Kyoto Encyclopedia of Genes and Genomes (KEGG) pathway associated with composite target genes. BPs, biological processes; CCs, cellular components; MFs, molecular functions.

**FIGURE 4:**
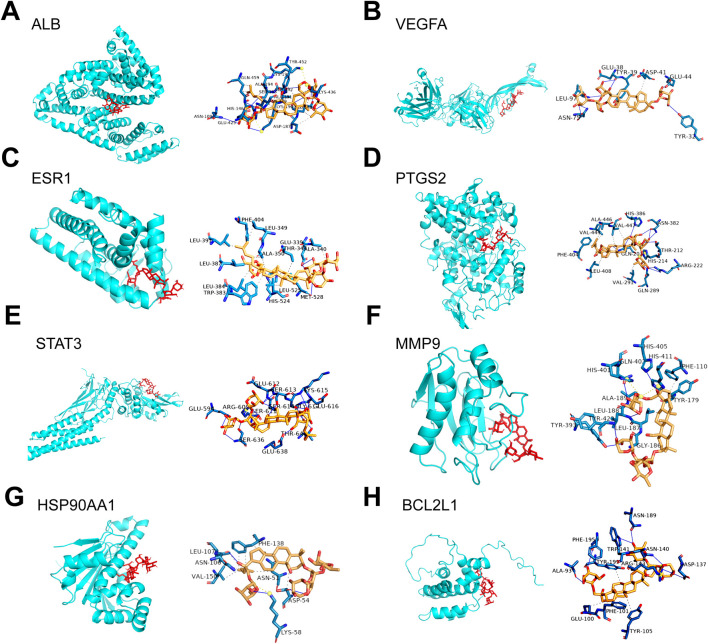
Molecular docking of Gyp XLIX to hub genes. Molecular docking of Gyp XLIX to ALB **(A)**, VEGFA **(B)**, ESR1 **(C)**, PTGS2 **(D)**, STAT3 **(E)**, MMP9 **(F)**, HSP90AA1 **(G)**, and BCL2L1 **(H)**.

A total of 136 non-redundant Gyp XLIX targets were obtained from PharmMapper and SwissTargetPrediction. 56 DEGs were identified between healthy individuals and patients with DR in the GSE53257 dataset, comprising 25 upregulated and 31 downregulated genes. A volcano map was generated to visualize the top 20 DEGs (Figure 1A), whereas a heat map was generated to visualize the upregulated and downregulated genes (Figure 1B). Furthermore, a total of 3,943, 188, and 645 DR-related genes were obtained from the GeneCards, OMIM, and DisGeNET databases, respectively. After comprehensive integration and deduplication, 4224 DR-related genes were retained for further analysis. The 136 potential targets of Gyp XLIX were intersected with the 4,224 DR-related targets, resulting in the identification of 66 common targets (Figure 2A). A PPI network comprised 63 nodes and 1,097 edges (Figure 2B). A total of 10 hub genes (ALB, VEGFA, JUN, ESR1, PTGS2, STAT3, MMP9, HSP90AA1, BCL2L1, AR) were identified. These genes had the highest degree values in the network (Figure 2C). GO analysis revealed that the hub genes were enriched in a total of 2,672 GO items, with the top 10 biological processes (BPs), cellular components (CCs), and molecular functions (MFs) shown, respectively (Figures 3A–C). The hub genes were enriched in 75 pathways, as revealed by KEGG pathway analysis. The 20 most significantly enriched pathways included hypoxia inducible factor-1 (HIF-1) signalling, VEGF signalling, inflammatory mediator regulation of transient receptor potential (TRP) channels, advanced glycation end products–receptor for advanced glycation end products (AGE-RAGE) signalling in diabetic complications, gap junction, etc. (Figure 3D). Molecular docking was performed to investigate the interactions between Gyp XLIX and the 10 hub genes (Figure 4). The binding free energy scores revealed strong binding of PTGS2 to Gyp XLIX, whereas JUN and AR displayed weaker affinities (Table 2). These lower scores do not undermine the PTGS2-centred model, given its functional centrality in the network and robust docking interaction.

**TABLE 2:** Molecular docking of Gyp XLIX to ligands.

Ligand	PDB ID	Binding energy score
ALB	1E7A	−8.2
VEGFA	1BJ1	−5.8
JUN	5FV8	−4.7
ESR1	1ERR	−6.4
PTGS2	1CVU	−7.0
STAT3	6NJS	−7.4
MMP9	2OVX	−6.4
HSP90AA1	1OSF	−6.2
BCL2L1	1YSI	−8.3
AR	1GS4	−4.1

### 3.2 Gyp XLIX preserved TJ integrity and suppressed ferroptosis in mice with DM

**FIGURE 5:**
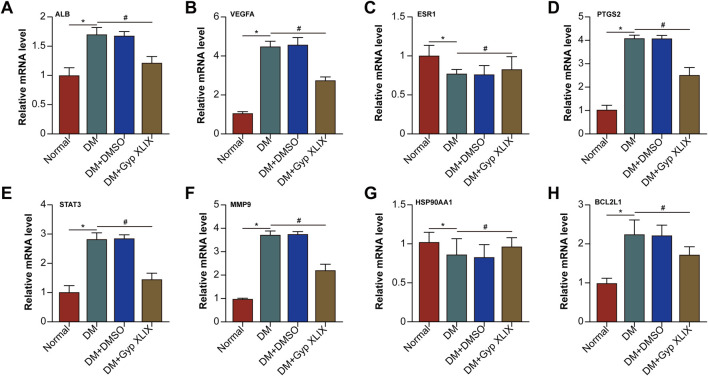
Expression changes of core targets. The effects of Gyp XLIX on the mRNA expression of ALB **(A)**, VEGFA **(B)**, ESR1 **(C)**, PTGS2 **(D)**, STAT3 **(E)**, MMP9 **(F)**, HSP90AA1 **(G)**, and BCL2L1 **(H)** were assessed by quantitative reverse transcription polymerase chain reaction (qRT-PCR). All data are presented as mean ± standard deviation (SD) from three independent biological replicates (n = 3), each measured in duplicate technical replicates. Statistical comparisons were performed using one-way ANOVA, with variance homogeneity evaluated using Brown–Forsythe and Bartlett’s tests. When the assumption of equal variances was violated, appropriate variance-corrected ANOVA methods were applied. Statistical significance was defined as ^*^p < 0.05, ^**^p < 0.01 versus the control group, ^#^p < 0.05, ^##^p < 0.01 versus the model group.

**FIGURE 6:**
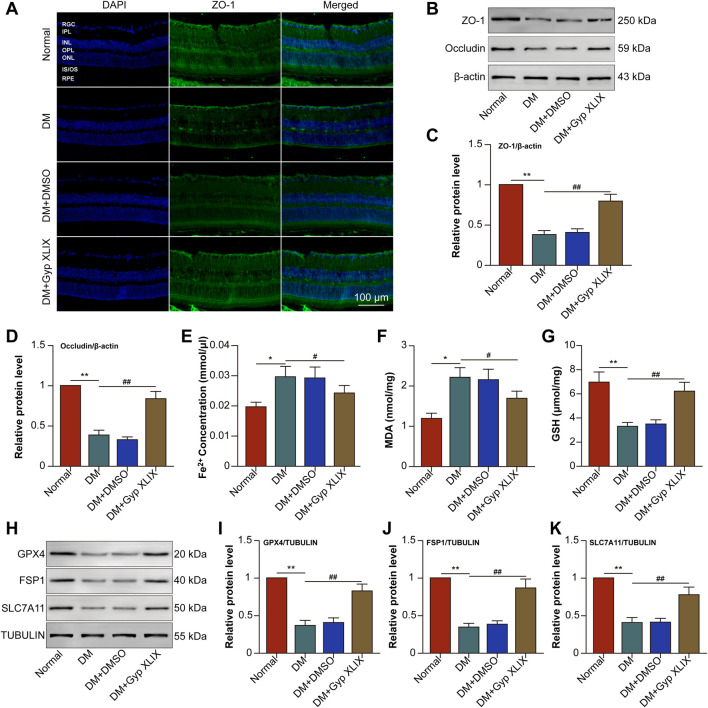
Gyp XLIX preserved tight junctions (TJ) integrity and suppressed ferroptosis in mice with diabetes mellitus (DM). **(A)** The effects of Gyp XLIX on zonula occludens-1 (ZO-1) expression were investigated by immunofluorescence staining, with cell nuclei being stained with DAPI. ZO-1 is represented by green fluorescence. **(B–D)** The effects of Gyp XLIX on the protein expression of ZO-1 and occludin were assessed by Western blotting. **(E–G)** The effects of Gyp XLIX on Fe^2+^, malondialdehyde (MDA) and glutathione (GSH) levels were examined by respective assay kits. **(H–K)** The effects of Gyp XLIX on the expression of glutathione peroxidase 4 (GPX4), ferroptosis suppressor protein-1 (FSP1) and solute carrier family 7 member 11 (SLC7A11) were assessed by Western blotting. RGC: retinal ganglion cell; IPL: inner plexiform layer; INL: inner nuclear layer; OPL: outer plexiform layer; ONL: outer nuclear layer; IS/OS: inner/outer segment; RPE: retinal pigment epithelium. All data are presented as mean ± standard deviation (SD) from three independent biological replicates (n = 3), each measured in duplicate technical replicates. Statistical comparisons were performed using one-way ANOVA, with variance homogeneity evaluated using Brown–Forsythe and Bartlett’s tests. When the assumption of equal variances was violated, appropriate variance-corrected ANOVA methods were applied. Statistical significance was defined as ^*^p < 0.05, ^**^p < 0.01 versus the control group, ^#^p < 0.05, ^##^p < 0.01 versus the model group.

Based on the above network pharmacological analysis, many potential mechanisms by which Gyp XLIX affects DR were identified. Retinal histopathological examination showed that successful induction of DR. 50 mg/kg significantly alleviated retinal damage caused by diabetes, while 100 mg/kg provided no additional benefit. Body weight and fasting plasma glucose remained stable across all doses, with no signs of toxicity (Supplementary Figure S1). Thus, 50 mg/kg was chosen as the optimal dose for subsequent studies. QRT-PCR analysis further revealed changes in core target gene expression following Gyp XLIX treatment in the RPE/choroid complexes of diabetic mice (Figure 5). Notably, PTGS2 mRNA levels were significantly elevated, whereas Gyp XLIX administration reversed this trend. PTGS2, also known as cyclooxygenase-2 (COX-2), is a critical biomarker strongly upregulated by lipid peroxidation products during ferroptosis. This process may subsequently lead to the dysregulation of TJ protein expression. Therefore, we evaluated the expression of ZO-1 in the retina-RPE-choroid tissues of mice with DM. The DM group showed significantly lower expression of ZO-1 than the normal control group, which was prevented by Gyp XLIX treatment (Figure 6A). Treatment with Gyp XLIX considerably increased the expression of ZO-1 and occludin in the RPE/choroid complexes of mice with DM (Figures 6B–D). Concurrently, we examined Fe^2+^, MDA, and GSH levels in the RPE/choroid complexes of mice with DM. As expected, the RPE/choroid complexes of the mice with DM exhibited lipid peroxidation, which was reflected by increased levels of Fe^2+^ and MDA and decreased levels of GSH. However, treatment with Gyp XLIX suppressed lipid peroxidation in mice with DM (Figures 6E–G). Furthermore, the RPE/choroid complexes of mice with DM had decreased protein expression of GPX4, FSP-1, and SLC7A11, indicating the occurrence of ferroptosis; however, treatment with Gyp XLIX reversed this downregulation (Figures 6H–K). These results collectively indicated that Gyp XLIX effectively inhibited ferroptosis and preserved TJ integrity in the RPE/choroid complexes of mice with DM.

### 3.3 Gyp XLIX inhibited HG-induced ferroptosis and loss of TJ proteins in ARPE-19 cells

**FIGURE 7:**
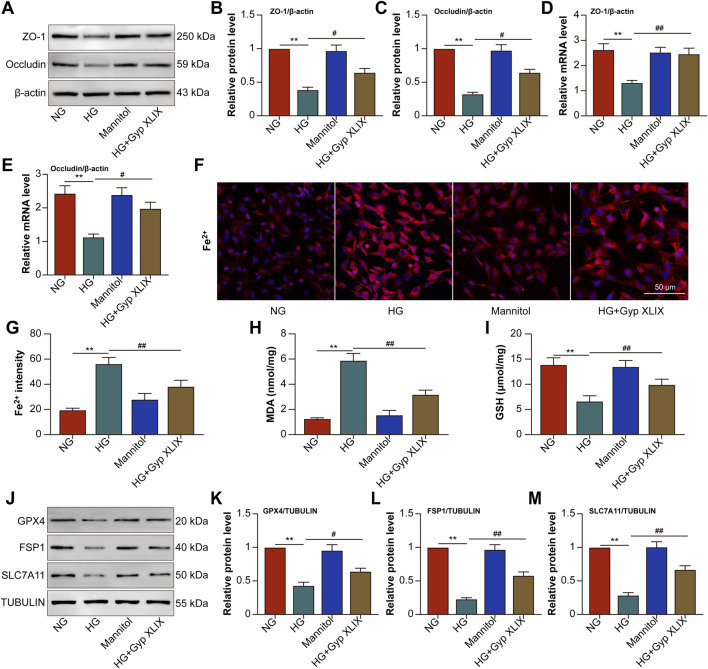
Gyp XLIX inhibited high glucose (HG)-induced ferroptosis and loss of epithelial junction proteins in ARPE-19 cells. **(A–C)** The effects of Gyp XLIX on the protein expression of zonula occludens-1 (ZO-1) and occludin were assessed by Western blotting. **(D,E)** The effects of Gyp XLIX on the mRNA expression of ZO-1 and occludin were assessed by quantitative reverse transcription polymerase chain reaction (qRT-PCR). **(F,G)** Fluorescence imaging of Fe^2+^ in living cells was performed with FerroOrange. **(H,I)** The effects of Gyp XLIX on malondialdehyde (MDA) and glutathione (GSH) levels were examined by respective assay kits. **(J–M)** The effects of Gyp XLIX on the protein expression of glutathione peroxidase 4 (GPX4), ferroptosis suppressor protein-1 (FSP1) and solute carrier family 7 member 11 (SLC7A11) were assessed by Western blotting. All data are presented as mean ± standard deviation (SD) from three independent biological replicates (n = 3), each measured in duplicate technical replicates. Statistical comparisons were performed using one-way ANOVA, with variance homogeneity evaluated using Brown–Forsythe and Bartlett’s tests. When the assumption of equal variances was violated, appropriate variance-corrected ANOVA methods were applied. Statistical significance was defined as ^**^p < 0.01 versus the control group, ^#^p < 0.05, ^##^p < 0.01 versus the model group.

We treated ARPE-19 cells with high-concentration glucose to replicate the pathological milieu of DR. Firstly, a cell viability assay with ARPE-19 was performed, which showed that Gyp XLIX at 10, 25, and 50 μmol/L did not cause any significant cytotoxicity compared to the control group, while concentrations of 100 and 200 μmol/L caused a decrease in cell viability. The concentration of 50 μmol/L was used for follow-up experiments. HG stimulation decreased the mRNA and protein expression of ZO-1 and occludin, whereas Gyp XLIX treatment reversed these changes (Figures 7A–E). Treatment with Gyp XLIX led to a reduction in the fluorescence intensity of Fe^2+^ (Figures 7F,G) and significantly increased GSH levels and decreased MDA levels (Figures 7H,I) in HG-stimulated ARPE-19 cells. In addition, it reversed the HG-induced decrease in the protein expression of GPX4, FSP-1, and SLC7A11 (Figures 7J–M). Altogether, these results suggested that HG stimulation decreased expression of TJ proteins, triggered lipid peroxidation, and promoted ferroptosis in ARPE-19 cells; however, treatment with Gyp XLIX effectively counteracted these changes.

### 3.4 Gyp XLIX inhibited HG-induced ferroptosis by downregulating PTGS2 in ARPE-19 cells

**FIGURE 8:**
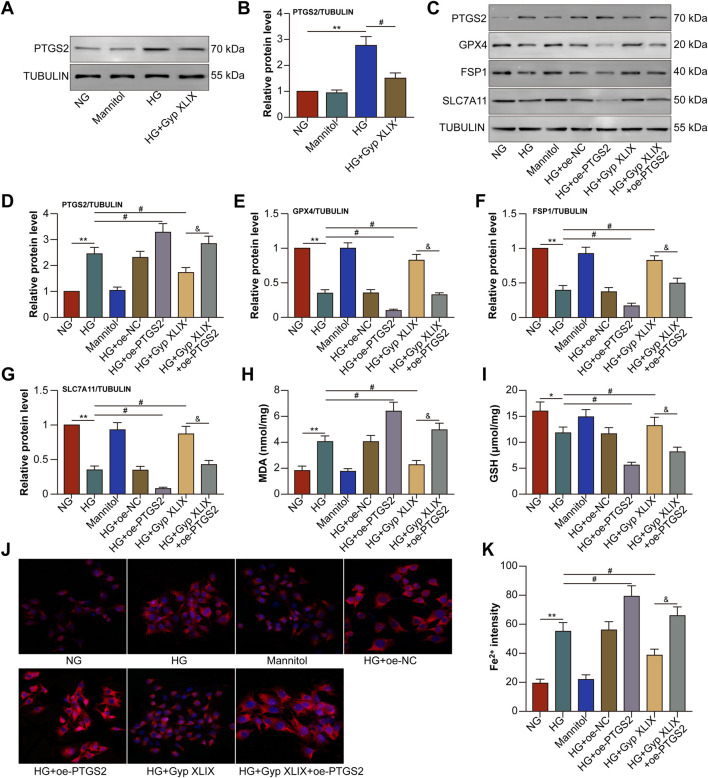
Gyp XLIX inhibited high glucose (HG)-induced ferroptosis by Downregulating prostaglandin-endoperoxide synthase 2 (PTGS2) in ARPE-19 cells. **(A,B)** The effects of Gyp XLIX on the protein expression of PTGS2 were assessed by Western blotting. **(C–G)** The effects of Gyp XLIX and oe-PTGS2 on the protein expression of PTGS2, glutathione peroxidase 4 (GPX4), ferroptosis suppressor protein-1 (FSP1) and solute carrier family 7 member 11 (SLC7A11) were assessed by Western blotting. **(H,I)** The effects of Gyp XLIX and oe-PTGS2 on malondialdehyde (MDA) and glutathione (GSH) levels were examined by respective assay kits. **(J,K)** The effects of Gyp XLIX and oe-PTGS2 on Fe^2+^ levels were assessed by FerroOrange. All data are presented as mean ± standard deviation (SD) from three independent biological replicates (n = 3), each measured in duplicate technical replicates. Statistical comparisons were performed using one-way ANOVA, with variance homogeneity evaluated using Brown–Forsythe and Bartlett’s tests. When the assumption of equal variances was violated, appropriate variance-corrected ANOVA methods were applied. Statistical significance was defined as ^*^p < 0.05, ^**^p < 0.01 versus the control group, ^#^p < 0.05 versus the model group, ^&^p < 0.05 versus the HG + Gyp XLIX group.

Consistent with *in vivo* observations, we found that the expression of PTGS2 was increased in the HG group and could been reversed by the treatment with Gyp XLIX (Figures 8A,B). To assess whether Gyp XLIX inhibited HG-induced ferroptosis by downregulating PTGS2, we transfected ARPE-19 cells with oe-NC or oe-PTGS2 before treating them with Gyp XLIX. Treatment with Gyp XLIX effectively increased the expression of GPX4, FSP-1, and SLC7A11 in HG-treated ARPE-19 cells; however, overexpression of PTGS2 attenuated these effects of Gyp XLIX (Figures 8C–G). In addition, overexpression of PTGS2 reversed the Gyp XLIX-induced increase in GSH levels and decrease in MDA levels in HG-treated ARPE-19 cells (Figures 8H,I). Similarly, overexpression of PTGS2 reversed the Gyp XLIX-induced attenuation of the fluorescence intensity of Fe^2+^ in HG-treated ARPE-19 cells (Figures 8J,K). These results collectively suggested that Gyp XLIX inhibited HG-induced ferroptosis by downregulating PTGS2 in ARPE-19 cells.

### 3.5 Gyp XLIX inhibited ferroptosis and preserved TJ integrity by downregulating PTGS2 in mice with DM

**FIGURE 9:**
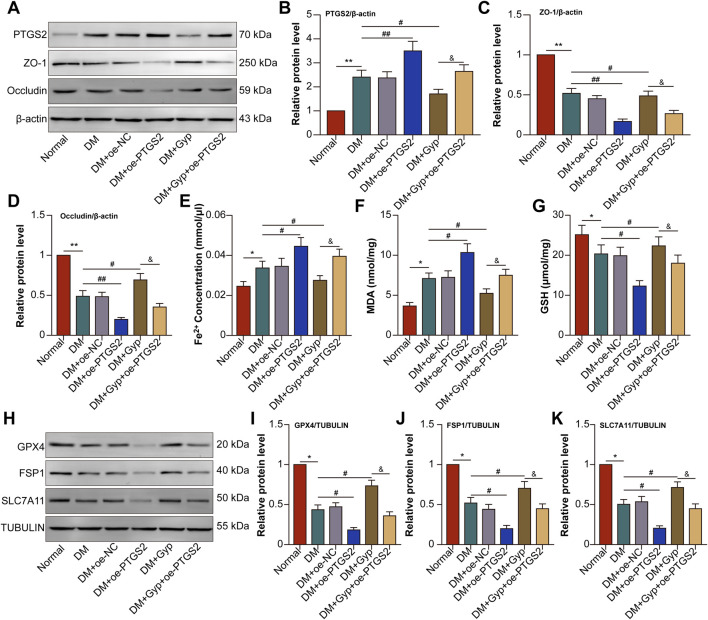
Gyp XLIX inhibited ferroptosis and preserved tight junctions (TJ) integrity by downregulating prostaglandin-endoperoxide synthase 2 (PTGS2) in mice with diabetes mellitus (DM). **(A–D)** The effects of Gyp XLIX and oe-PTGS2 on the protein expression of PTGS2, zonula occludens-1 (ZO-1), and occludin were assessed by Western blotting. **(E–G)** The effects of Gyp XLIX and oe-PTGS2 on Fe^2+^, malondialdehyde (MDA) and glutathione (GSH) levels were examined by respective assay kits. **(H–K)** The effects of Gyp XLIX and oe-PTGS2 on the protein expression of glutathione peroxidase 4 (GPX4), ferroptosis suppressor protein-1 (FSP1) and solute carrier family 7 member 11 (SLC7A11) were assessed by Western blotting. All data are presented as mean ± standard deviation (SD) from three independent biological replicates (n = 3), each measured in duplicate technical replicates. Statistical comparisons were performed using one-way ANOVA, with variance homogeneity evaluated using Brown–Forsythe and Bartlett’s tests. When the assumption of equal variances was violated, appropriate variance-corrected ANOVA methods were applied. Statistical significance was defined as ^*^p < 0.05, ^**^p < 0.01 versus the control group, ^#^p < 0.05, ^##^p < 0.01 versus the model group, ^&^p < 0.05 versus the HG + Gyp XLIX group.

Subsequently, we investigated whether Gyp XLIX attenuates ferroptosis and preserves TJ integrity by regulating PTGS2 *in vivo*. The results revealed that PTGS2 was effectively overexpressed and that the overexpression of PTGS2 reversed the Gyp XLIX-induced increase in the protein expression of ZO-1 and occludin in the RPE/choroid complexes of mice with DM, indicating that Gyp XLIX exerted protective effects on TJ integrity through PTGS2 (Figures 9A–D). In addition, overexpression of PTGS2 partially reversed the Gyp XLIX-induced decrease in Fe^2+^ and MDA levels and increase in GSH levels in the RPE/choroid complexes of mice with DM (Figures 9E–G). Similarly, overexpression of PTGS2 counteracted the effects of Gyp XLIX on the protein expression of GPX4, FSP-1, and SLC7A11 in the RPE/choroid complexes of mice with DM (Figures 9H–K). These results suggested that Gyp XLIX inhibited ferroptosis and preserved TJ integrity by downregulating PTGS2 in mice with DM.

## 4 Discussion

DR is a chronic and progressive complication of DM, and the mechanisms underlying its pathogenesis remain unclear. A newly developed microscopic imaging assay revealed that breakdown of the RPE barrier, associated with occludin depletion, leads to oBRB leakage in diabetic and ischemic rodents, highlighting the significance of TJs in RPE cells in ocular diseases like DME (Xu and Le, 2011). In this study, we used network pharmacology and experimental validation to investigate the early protection of junctional integrity by Gyp XLIX in DR and the associated mechanism. Gyp XLIX confers a protective effect against DR by acting on 8 proteins, namely, ALB, VEGFA, ESR1, PTGS2, STAT3, MMP9, HSP90AA1 and BCL2L1 through network pharmacological analysis. In addition, preliminary *in vivo* and *in vitro* experiments verified that Gyp XLIX inhibited ferroptosis and preserved TJ integrity in early DR, effects that were associated with downregulation of PTGS2.

Modern phytochemical and pharmacological studies have substantially validated that *G. pentaphyllum* is rich in dammarane-type saponins (gypenosides) structurally similar to ginsenosides, which exhibit a wide range of bioactivities including anti-cancer (Liu H. et al., 2022), anti-inflammatory (Huang et al., 2022), anti-viral (Yang G. et al., 2021), hepatoprotective (Wang et al., 2021), cardioprotective (Su et al., 2022), and neuroprotective (Zhai et al., 2021) activities. These activities align remarkably well with its traditional uses against inflammatory and metabolic disorders. This convergence of traditional wisdom and modern scientific evidence provided the fundamental rationale for selecting *G. pentaphyllum*, and specifically its major saponin component Gyp XLIX, for our investigation into potential therapeutic mechanisms against DR. Gypenosides share structural similarity with ginsenosides and notoginsenosides, which are biologically active compounds identified in the roots of *Panax ginseng* and *Panax notoginseng*, respectively (Lou et al., 2021; Wang et al., 2020). A recent study showed that gypenoside A decreased blood glucose levels and alleviated pancreatic β-cell dysfunction by targeting microRNA-150-3p (MiR-150-3p), thus exerting therapeutic effects against DM (Li, 2022). The effects of gypenoside LXXV, a novel natural agonist of PPAR-ɣ, on cognitive deficits have been investigated in db/db mice. Mechanistically, gypenoside LXXV increases glucose uptake in the brain through the activation of protein kinase B (PKB)/glucose transporter 4 (GLUT4) signaling, thereby improving cognitive function (Meng et al., 2022). Gypenoside XVII ameliorates early DR in db/db mice by modulating Müller cell apoptosis and autophagy (Luo et al., 2021). Gyp XLIX, a selective activator of PPAR-α (Huang et al., 2007), plays a protective role in acute kidney injury by suppressing insulin-like growth factor-binding protein 7 (IGFBP7)/insulin-like growth factor 1 receptor (IGF1R)-mediated programmed cell death and inflammation (Yang Q. et al., 2021). In this study, we found that Gyp XLIX exerted early protective effects on junctional integrity in mice with DM. In the retina of patients with diabetes, depletion of occludin and damage to the TJs of RPE cells result in oBRB disruption, which is a key factor in DME, the most prevalent cause of vision impairment in DR (Talu and Nicoara, 2021). Therefore, Gyp XLIX may alleviate visual impairment in patients with DR by protecting TJs disruption in RPE cells. It should be noted that while ZO-1 and occludin are well-established markers of tight junction integrity, their expression levels alone do not fully recapitulate barrier function. Future studies incorporating functional measurements such as transepithelial electrical resistance (TEER) or paracellular flux assays will be valuable to directly confirm the protective effects of Gyp XLIX on RPE barrier function.

To investigate the therapeutic mechanism of Gyp XLIX against DR, 66 common targets of Gyp XLIX and DR were identified via network pharmacology. Among these targets, ALB, VEGFA, JUN, ESR1, PTGS2, STAT3, MMP9, HSP90AA1, BCL2L1, and AR were identified as hub genes, suggesting that Gyp XLIX can be utilised as a therapeutic agent for DR through a multi-target approach. Multiple clinical trials have demonstrated the efficacy of intravitreal injection of anti-VEGF agents in the treatment of DR. This treatment modality has been adopted on a global scale (Ferrara and Adamis, 2016). The therapeutic effects of anti-VEGF agents are attributed to the role of VEGFA in both angiogenesis and vascular permeability. Additionally, researches have shown that long noncoding RNAs (lncRNAs) can suppress HG-induced dysfunction of retinal vascular endothelial cells by interacting with signal transducer and activator of transcription 3 (STAT3) and target of VEGFA (Sun et al., 2021). In DR, the c-Jun kinase (c-Jun)/tissue inhibitor of metalloproteinase-3 (TIMP-3)/tumour necrosis factor-alpha (TNF-α)-converting enzyme (TACE) pathway is inhibited, which in turn inhibits TNF-α production and retinal microvascular endothelial cell apoptosis, offering potential targets for DR (Zhang and Steinle, 2014). The core-binding factor subunit beta (CBF-β)/estrogen receptor 1 (ESR1) axis regulates the expression of Trefoil factor family 1 (Tff1), thereby suppressing retinal endothelial cell proliferation and angiogenesis (Zhang et al., 2022). In addition to endothelial dysfunction and angiogenesis, the therapeutic efficacy of corticosteroids in DME fully illustrates the significance of inflammation in the progression of DR (Rittiphairoj et al., 2020). Furthermore, matrix metalloproteinase-9 (MMP9) can accelerate the apoptosis of retinal capillary cells (Mohammad and Kowluru, 2012). In a study, proteomic analysis revealed increased expression of heat shock protein 90-alpha (HSP90AA1) in the macular region of healthy donors. This finding suggests that specific retinal regions may be vulnerable to distinct forms of metabolic and oxidative stress (Velez et al., 2018). A recent study revealed that PTGS2 ameliorated ferroptosis-mediated myocardial injury and inflammation in coronary microembolization (Liu T. et al., 2022). Given the prevailing knowledge regarding the hub genes, we speculate that Gyp XLIX protects against DR by suppressing inflammation, apoptosis, neovascularization and vascular leakage. GO analysis demonstrated that the hub genes were highly enriched in positive regulation of signalling, nuclear receptor activity and signalling receptor binding. In addition, KEGG analysis revealed that the hub genes were associated with the HIF-1 and VEGF signalling pathways, inflammatory mediator regulation of TRP channels, and the AGE–RAGE signalling pathway in diabetic complications. These findings indicate that the key targets of Gyp XLIX identified in this study are associated with various pathological processes of DR. Among the numerous enriched signaling pathways, there is a strong correlation between the HIF-1 signaling pathway and PTGS2. In renal cancer cells and gastric cancer cells, HIF serves as a key driver of ferroptosis. Additionally, HIF-2α significantly upregulates lipid/iron metabolism-related genes in colorectal cancer, thereby priming cells for ferroptosis (Jin et al., 2024). Naringin (NG), a naturally occurring flavanone glycoside, attenuates inflammation in RPE cells by modulating the heme oxygenase-1 (HO-1)/GPX4 pathway to suppress ferroptosis (Yang et al., 2025). Our research results indicate that Gyp XLIX inhibits HG-induced ferroptosis in RPE cells by downregulating PTGS2, potentially mediated through HIF-1 signaling pathway, warranting further investigation. Therefore, the results of network pharmacology may serve as a valuable reference for further investigation into the pathogenesis of DR.

Ferroptosis has attracted extensive interest in ophthalmology. Abnormal iron accumulation has been detected in the vitreous samples from patients with DR and the retinas of animal models of STZ-induced DR (Chaudhary et al., 2018; Konerirajapuram et al., 2004). Iron overload in patients with DR may involve the following mechanisms: first, hyperglycaemia destroys heme and releases free iron; second, increased angiotensin (Ang)-II levels stimulate the expression of genes involved in iron metabolism, thereby promoting iron accumulation (Ishizaka et al., 2007). *In vitro* evidence indicates that ferroptosis in RPE cells induced by HG contributes to the development of DR. The possible mechanisms involve the miR-338-3p/solute carrier family 1 member 5 and miR-138-5p/sirtuin 1/nuclear factor erythroid 2-related factor 2 (Nrf2) pathways. However, further experimental data are still required (Tang et al., 2022; Zhou et al., 2022). Ferroptosis of RPE cells can result in oBRB disruption, consequently inducing DME (Chaudhary et al., 2018). In this study, we found that treatment with Gyp XLIX decreased Fe^2+^ and MDA levels and increased GSH levels in mice with DM. Furthermore, Gyp XLIX treatment upregulated key ferroptosis regulators (GPX4, FSP-1, and SLC7A11) in mice with DM. Altogether, Gyp XLIX inhibited ferroptosis, thereby exerting early protective effects on junctional integrity in mice with DR. These findings were validated in HG-stimulated ARPE-19 cells. Based on the results of network pharmacology screening and experimental verification, we focused our research on one of the core targets, namely, PTGS2, which is a ferroptosis-related protein. Ferroptosis is critically linked to dysregulated arachidonic acid metabolism and inflammatory cascades. PTGS2 (encoding the inducible enzyme COX-2) catalyzes the conversion of arachidonic acid to prostaglandin G_2_ (PGG_2_) and prostaglandin H_2_ (PGH_2_), generating diverse bioactive inflammatory mediators. Elevated PTGS2 expression is recognized as a ferroptosis marker and promotes arachidonic acid metabolism, thereby amplifying inflammatory mediator release. In recent years, bioinformatics, multi-omics and experimental validation have revealed that PTGS2 is a key gene associated with ferroptosis (Gao et al., 2024; Yuan et al., 2024). A recent study demonstrated that the Nrf2/GPX4/PTGS2 axis is involved in the anti-ferroptosis mechanism of resveratrol in retinal Müller cells (Wang et al., 2024). Our findings indicated that Gyp XLIX decreased the expression of PTGS2 in mice with DM and HG-stimulated ARPE-19 cells. Furthermore, overexpression of PTGS2 partially reversed the Gyp XLIX-induced inhibition of ferroptosis and preservation of TJ integrity, which indicated that Gyp XLIX protected against DR in part by downregulating PTGS2 to suppress ferroptosis. Although PTGS2 is upregulated during ferroptosis and its inhibition correlates with protection, it remains possible that PTGS2 serves as a sensitive biomarker of ferroptotic stress rather than an essential initiating mediator. While PTGS2 is a sensitive marker of ferroptosis, its functional role requires further investigation using genetic or pharmacological loss-of-function approaches. Moreover, measurement of downstream eicosanoids such as PGE_2_ would be necessary to confirm that the observed changes in PTGS2 protein reflect altered enzymatic activity. It is helpful to clarify the relationship between PTGS2 and the eicosanoid axis within the current research scope.

Despite significant findings, this study has some limitations that merit consideration. First, we focused on expression alterations of core targets in the RPE/choroid complexes, identifying a novel candidate for preserving TJ integrity of DR. However, parallel alterations of core targets in the neural retina tissues have sparked considerable investigative interest. As a multifaceted disorder engaging neuro-vascular-immune-metabolic networks, DR demands deeper mechanistic exploration and integration to unravel its systemic pathophysiology. Second, we focused on only one of the core targets, namely, PTGS2, the roles of other core targets were not investigated. In future studies, we will further examine the roles of other core targets in the pathogenesis of DR, and validate the multi-target protective effects of Gyp XLIX against DR. Furthermore, while our data support early protection of tight junctional integrity of DR, further long-term studies are required to determine whether this early protection can prevent advanced DR.

## 5 Conclusion

**FIGURE 10:**
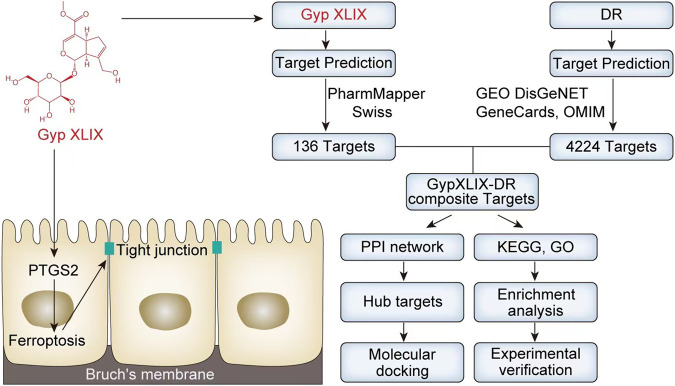
This study revealed a potential target of Gyp XLIX for early protection of junctional integrity of diabetic retinopathy (DR). The findings of this study preliminarily suggest that Gyp XLIX inhibits ferroptosis and preserves tight junctions (TJ) integrity by downregulating prostaglandin-endoperoxide synthase 2 (PTGS2), thus playing an early protective role in junctional integrity of DR.

In conclusion, this study revealed a potential target of Gyp XLIX for early protection of junctional integrity of DR. The findings of this study preliminarily demonstrate that Gyp XLIX inhibits ferroptosis and preserves TJ integrity by downregulating PTGS2, thus playing an early protective role in junctional integrity of DR (Figure 10). This study provides a theoretical and experimental reference for the subsequent clinical implementation of Gyp XLIX in the treatment of DR. Further studies are warranted to verify the therapeutic efficacy of Gyp XLIX in DR through other core targets.

## Data Availability

The original contributions presented in the study are included in the article/Supplementary Material, further inquiries can be directed to the corresponding authors.
