# Supplementary material for: Gypenoside XLIX ameliorates diabetic retinopathy by downregulating prostaglandin-endoperoxide synthase 2 in retinal pigment epithelium cells to inhibit ferroptosis and preserve tight junction integrity
Source: Front Pharmacol. 2026 Mar 23;17:1777313. doi: 10.3389/fphar.2026.1777313 (PMC13050865; doi:10.3389/fphar.2026.1777313)
Supplement: Supplementary file 1 [file Presentation1.pdf]

Supplementary Material

1 Supplementary Figures and Tables

1.1 Supplementary Figures

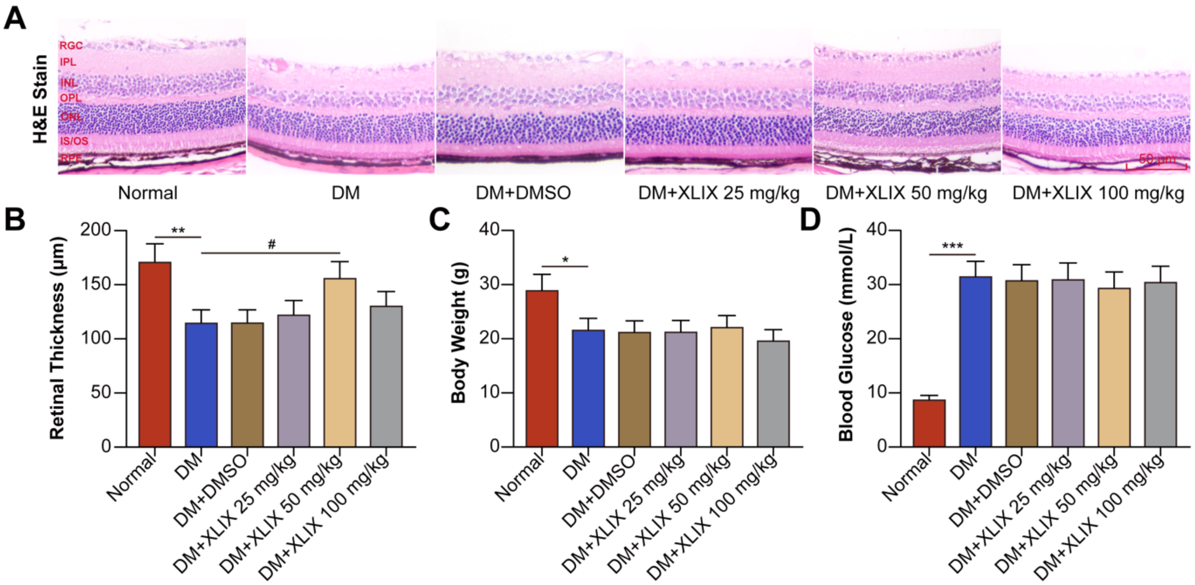

Supplementary Figure 1. (A) HE staining of retinal tissue was performed at week 8. RGC, retinal ganglion cell layer; IPL, inner plexiform layer; INL, inner nuclear layer; OPL, outer plexiform layer; ONL, outer nuclear layer; IS/OS, inner segment/outer segment; RPE, retinal pigment epithelium. (B) Quantification of the total retinal thickness in the circular area around the optic nerve head was performed. (C) Mouse body weight was measured at week 8. (D) Mouse fasting plasma glucose levels were measured at week 8. All data are presented as mean ± standard deviation (SD) from six independent biological replicates (n = 6), each measured in duplicate technical replicates. Statistical comparisons were performed using one-way ANOVA, with variance homogeneity evaluated using Brown–Forsythe and Bartlett's tests. When the assumption of equal variances was violated, appropriate variance-corrected ANOVA methods were applied. Statistical significance was defined as $^{*}p < 0.05$, $^{**}p < 0.01$, $^{***}p < 0.001$ versus the normal group, $^{\#}p < 0.05$ versus the model group.

1.2 Supplementary Tables

Supplementary Table 1. Information of the network section.

Database/Tool	Version/Access Date	Filtering Criteria/Parameters	Deduplication Rules
PubChem (https://pubchem.ncbi.nlm.nih.gov/)	Accessed 2022	Default query	-
PharmMapper (http://lilab.ecust.edu.cn/pharmMapper/)	Updated 2022	Default parameters	UniProt ID, retain only Swiss-Prot entries
SwissTargetPrediction (http://www.swisstargetprediction.ch/)	Accessed 2022	Species = *Homo sapiens*	UniProt ID, retain only Swiss-Prot entries
Gene Expression Omnibus (GEO) database (http://www.ncbi.nlm.nih.gov/geo) (GSE53257)	Accessed 2022	Adjusted $P < 0.05$, $\lvert \log_2 FC \rvert > 0.5$	-
GeneCards (https://www.genecards.org/)	Version 5.20	Relevance score ≥ 1.0	UniProt ID, retain only Swiss-Prot entries
OMIM (https://omim.org/)	Accessed 2022	Morbid map only	UniProt ID, retain only Swiss-Prot entries
DisGeNET databases (http://www.disgenet.org/)	Version 7.0	Score ≥ 0.1	UniProt ID, retain only Swiss-Prot entries
STRING (https://string-db.org/)	Version 11.5	Confidence score ≥ 0.7	-
Cytoscape	Version 3.7.2	-	-

Supplementary Table 1. Continued table.

Database/Tool	Version/Access Date	Filtering Criteria/Parameters	Deduplication Rules
R package org.Hs.eg.db	Version 3.1.0	-	-
KEGG REST API (https://www.kegg.jp/kegg/rest/keggapi.html)	Accessed 2022	-	-
R package clusterProfiler	Version 3.14.3	$P < 0.05$, FDR < 0.25	-
Protein Data Bank (PDB)	Accessed 2022	Resolution ≤ 3.0 Å	-
PyMOL	Version 2.5.2	Dehydration, ligand removal, complex visualization	-
AutoDock Vina	Version 1.2.3	Binding energy < –5.0 kcal/mol	-
